# Oxidative Stress Induced by Lipotoxicity and Renal Hypoxia in Diabetic Kidney Disease and Possible Therapeutic Interventions: Targeting the Lipid Metabolism and Hypoxia

**DOI:** 10.3390/antiox12122083

**Published:** 2023-12-06

**Authors:** Seung Yun Chae, Yaeni Kim, Cheol Whee Park

**Affiliations:** 1 Division of Nephrology, Department of Internal Medicine, Seoul St. Mary’s Hospital, The College of Medicine, The Catholic University of Korea, 222, Banpo-daero, Seocho-gu, Seoul 06591, Republic of Korea; hippoc@catholic.ac.kr (S.Y.C.); 20800137@cmcnu.or.kr (Y.K.); 2 Institute for Aging and Metabolic Disease, Seoul St. Mary’s Hospital, The College of Medicine, The Catholic University of Korea, 222, Banpo-daero, Seocho-gu, Seoul 06591, Republic of Korea

**Keywords:** diabetic kidney disease, hypoxia, lipotoxicity, oxidative stress

## Abstract

Oxidative stress, a hallmark pathophysiological feature in diabetic kidney disease (DKD), arises from the intricate interplay between pro-oxidants and anti-oxidants. While hyperglycemia has been well established as a key contributor, lipotoxicity emerges as a significant instigator of oxidative stress. Lipotoxicity encompasses the accumulation of lipid intermediates, culminating in cellular dysfunction and cell death. However, the mechanisms underlying lipotoxic kidney injury in DKD still require further investigation. The key role of cell metabolism in the maintenance of cell viability and integrity in the kidney is of paramount importance to maintain proper renal function. Recently, dysfunction in energy metabolism, resulting from an imbalance in oxygen levels in the diabetic condition, may be the primary pathophysiologic pathway driving DKD. Therefore, we aim to shed light on the pivotal role of oxidative stress related to lipotoxicity and renal hypoxia in the initiation and progression of DKD. Multifaceted mechanisms underlying lipotoxicity, including oxidative stress with mitochondrial dysfunction, endoplasmic reticulum stress activated by the unfolded protein response pathway, pro-inflammation, and impaired autophagy, are delineated here. Also, we explore potential therapeutic interventions for DKD, targeting lipotoxicity- and hypoxia-induced oxidative stress. These interventions focus on ameliorating the molecular pathways of lipid accumulation within the kidney and enhancing renal metabolism in the face of lipid overload or ameliorating subsequent oxidative stress. This review highlights the significance of lipotoxicity, renal hypoxia-induced oxidative stress, and its potential for therapeutic intervention in DKD.

## 1. Introduction

Lipotoxicity, which results from the excessive accumulation of detrimental lipid intermediates in non-adipose tissue cells, was initially described as a specific dysfunction in pancreas β-cells resulting from obesity and type 2 diabetes mellitus (DM) [1]. Ordinarily, the surge in lipids is counterbalanced by their storage in adipose tissues and through various cellular pathways addressing lipids. However, when this compensatory capacity is surpassed by an overabundance of lipids, the system can no longer neutralize the effects of lipotoxicity. This improper buildup of lipids including free fatty acid (FFA)s and cholesterols in non-adipose tissues triggers a range of cellular malfunctions, encompassing organelle disturbances, persistent inflammation, cellular damage, and cell death. Notably, organs such as the pancreas, liver, heart, and kidney are frequently affected by lipotoxicity. Diabetes is widely recognized as a lipogenic state [2]. Numerous studies have identified lipotoxicity as a significant factor in the progression of diabetic kidney disease (DKD) [3]. Consequently, mitigating lipotoxicity could serve as an effective therapeutic strategy for this condition.

The kidney is a metabolically active organ, second only to the heart in terms of oxygen consumption divided by the mass of tissue. In fact, the kidneys receive roughly 20% of cardiac output. Despite this, the nature of the kidney necessitates a high demand for ATP, and since most of these ATPs are derived from oxidative phosphorylation [4], the organ is perpetually at risk of relative hypoxic injury [5]. A significant portion of the kidney’s ATP expenditure is attributed to the Na^+^/K^+^-ATPase pump [6]. This pump operates in the basolateral membrane, enabling various tubular transporters, including sodium–glucose co-transporter 2 (SGLT2), to utilize the electrochemical gradient between membranes for solute transport. Since one of the kidney’s principal functions is regulating sodium concentration through reabsorption and excretion, this high demand for ATP is expected [4]. Unfortunately, hypoxia causes the accumulation of macrophages in the kidney that produce profibrotic cytokines, such as transforming growth factor-ß (TGF-ß) and activate renal fibrosis [7].

Most chronic kidney disease (CKD) conditions are associated with hypoxic conditions linked to metabolic diseases, such as metabolic syndrome, obesity, and diabetes. A growing number of studies have linked oxidative stress with lipotoxicity and hypoxic damage in the diabetic condition. In this review, we focus on the molecular pathways of lipid accumulation and hypoxia within the kidney related to the diabetic condition. Additionally, we highlight the significance of lipotoxicity and renal hypoxia-induced oxidative stress, as well as its potential for therapeutic intervention in DKD.

In this review, we frequently cite studies utilizing diabetic animal models, which are diverse. To prevent confusion among readers, we have succinctly summarized the diabetic models employed in the cited research using a table for clarity and ease of reference. (Table 1).

## 2. Lipotoxicity and DKD

### 2.1. Lipid Metabolism—Physiologic Versus DKD

#### 2.1.1. Fatty Acid (FA) (Figure 1)

##### FA Uptake—Normal and DKD Patients

To fully understand the intracellular mechanisms governing lipids, it is crucial to examine how FAs, derived from our diet, are taken up by cells. While FAs have the capability to traverse the cellular membrane, they predominantly rely on transporters for this process. Notably, ① cluster of differentiation (CD)36, also known as FA translocase, is a scavenger receptor mainly tasked with facilitating the entry of long-chain FAs (LCFAs) into cells. FAs with 12 carbon atoms or more are referred to as LCFAs, while FAs with less than 12 carbon atoms are referred to as short-(SCFAs) and medium-chain FAs (MCFAs). FA chains may contain no double bonds (saturated) or one or more double bonds (unsaturated). Moreover, ② fatty acid-binding proteins (FABPs) and ③ fatty acid transport proteins (FATPs) play pivotal roles in the intracellular transportation of FAs. FABPs, of which there are nine subtypes categorized according to their cellular location, are lipid-binding proteins that assist in the transportation and utilization of FAs. For instance, L-FABP (FABP1) is predominantly located in the kidney’s proximal tubules, while A-FABP (FABP4) is present in peritubular capillaries [8]. FATPs, on the other hand, are transmembrane proteins, with six known subtypes (Solute Carrier Family SLC27A1-6) [9]. Among these, FATP2 is primarily expressed in kidney cells and aids in the uptake of non-ester fatty acids (NEFAs) [10]. Additionally, Acyl-CoA synthetase plays an indirect yet essential role in the uptake of FFAs [11]. This is because it sustains the concentration gradient of FFAs across the plasma membrane by attaching CoA to FFAs within the cell. This action promotes the influx of FFAs into cells via the aforementioned FA transporters, and the FFAs goes into the β-oxidation process.

Several studies have highlighted the significance of CD36 in DKD. While findings have occasionally varied [12], soluble CD36 has been proposed as a potential marker for insulin resistance in diabetes [13] and as a prognostic indicator for DKD [14]. Moreover, in mouse models of DKD, those with a *CD36* knockout (KO) demonstrated reduced renal fibrosis and improved renal function [15]. Therefore, the contribution of CD36 as a nexus between lipotoxicity and DKD warrants careful consideration. Within the family of FABPs, FABP1 has been observed to be downregulated in the kidneys of individuals with DKD [16]. Given that FABP1 plays a pivotal role in the utilization of FAs, its reduced expression might explain the lipid accumulation seen in the kidneys of DKD patients. Some studies have even posited that detecting FABP1 in urine could serve as an early diagnostic indicator for DKD [17]. Furthermore, FABP4 has been found to be upregulated in the mesangial cells [18] of DKD patients and in the podocytes [19] of DKD mouse models. FATP1, FATP2, and FATP4 have been identified as being potentially linked to challenges in lipid uptake among DKD patients [20].

##### FA Synthesis

Both glucose and glutamine lead to the generation of citrate. α-ketoglutarate evolved from glutaminolysis can also serve as a source of citrate. This citrate is subsequently split by ATP-citrate lyase (ACLY) into acetyl-CoA and oxaloacetate. ① The enzyme ACLY is activated through phosphorylation by Akt (protein kinase B). Additionally, acetyl-CoA can be synthesized from acetate, either absorbed from the surrounding environment or sourced intracellularly. Following this, ② acetyl-CoA undergoes carboxylation to form malonyl-CoA, which is then polymerized by ③ FA synthase (FASN) through a series of reactions to produce palmitate. This palmitate can be ④ further elongated by FA elongases (ELOVLs) and desaturated in the Δ9 position by stearoyl-CoA desaturases (SCDs). Additionally, other FA desaturases (FADSs) have the capability to introduce double bonds in position Δ5, Δ6, or Δ9 in LCFAs [21]. The expression of enzymes involved in such FA synthesis is regulated by ⑤ sterol regulatory element-binding proteins (SREBP)-1 [22].

As anticipated, the expression of SREBP and FASN is heightened in the glomeruli and tubular interstitium of diabetic kidneys. This observation is consistent in both DKD mouse models [23] and in mice with SREBP overexpression [24]. Nonetheless, given that downstream enzymes, such as FASN, exhibited reduced expression in DKD patients [16], the involvement of SREBP-1 in DKD is still controversial. Carbohydrate response element-binding protein (ChREBP) is also a transcription factor that is responsible for lipid metabolism. When it senses glucose, ChREBP induces lipid accumulation in the cells by enhancing lipid synthesis. ChREBP-KO *db*/*db* mice exhibited lower kidney lipid accumulation with attenuated mTOR activity [25].

##### FA Oxidation (FAO)

β-oxidation, often referred to as FAO, is the process in which FAs—whether they are short-, medium-, or long-chain saturated fatty acyl coenzyme A (acyl-CoA) varieties—are broken down aerobically within the mitochondria to derive energy from fats. β-oxidation predominantly occurs in the mitochondria, where FFAs are directed for breakdown. ① This process necessitates the activation of these FAs by binding them to CoA. ② Subsequently, the acyl group undergoes a transfer to carnitine palmitoyl transferase 1 (CPT1). Once transformed into acylcarnitine, it is relayed into the mitochondrial matrix via the actions of the carnitine–acylcarnitine translocase (CACT). During β-oxidation, energy is harnessed at multiple stages: each progressive reduction of acyl-CoA results in the production of one FADH_2_ and one NADH molecule every two carbon segments liberated. Every ③ acetyl-CoA molecule subsequently contributes to the generation of three NADH molecules, one FADH_2_ molecule, and one GTP molecule through the ④ tricarboxylic acid (TCA) cycle [21].

Reports have indicated the relationship between decreased β-oxidation and DKD. In an experiment utilizing mice with diabetes induced using streptozotocin (STZ), when PPAR-α, a key player in β-oxidation, was knocked out (KO), there was a noticeable increase in serum FAs. Additionally, compared to the wild-type diabetic-induced mice, the KO mice exhibited more severe symptoms, including albuminuria, glomerular sclerosis, and an expansion of the mesangial area [26]. In patients with DKD, the renal expression levels of proteins associated with mitochondrial biogenesis, peroxisome proliferative-activated receptor ɣ coactivator (PGC)-1α, and FAO (including PPAR-α and CPT-1) were found to be reduced compared to those in healthy individuals and those with type 2 DM [27]. Upon measuring the serum FAs of Native American patients diagnosed with DKD using the LC-MS technique and assessing the expression of the acetyl-CoA carboxylase gene in the kidney, a previously undetected connection that links lipid markers of compromised mitochondrial β-oxidation and augmented lipogenesis to DKD progression was identified [28].

⑤ Peroxisomes are instrumental in managing the breakdown of long-chain and very-long-chain fatty acyl-CoAs, dicarboxylic FAs, 2-methyl-branched FAs, and certain inflammatory mediators like eicosanoids, prostaglandins, and intermediate compounds of bile acids [29]. Additionally, the oxidation of exceptionally LCFA or branched FAs takes place within peroxisomes, leading to hydrogen peroxide formation. It is essential to note that peroxisomal β-oxidation only partially breaks down FAs since it does not directly contribute to the oxidative phosphorylation required for ATP production. PPARs stand out as key transcriptional overseers of this β-oxidation process within peroxisomes. As most of studies are focused on mitochondrial β-oxidation, more investigations are needed for elucidating the role of peroxisomal oxidation in DKD. However, diabetic mice with catalase KO were reported to show an increased proteinuria, attenuated renal function, increased serum FFAs, and increased mitochondrial reactive oxygen species (ROS) [30]. Also, in two types of DM mouse model, the hallmark proteins of peroxisomal oxidation were found to be decreased and this phenomenon was validated from the kidney tissues of DKD patients [31].

##### FA-Induced Post-Translational Modifications (PTMs)

PTM indicates the addition of biochemical groups to most proteins enzymatically or non-enzymatically, either during or after their translation, to control their structure, location, and/or activities. In various disease states, the impact and roles of PTM are increasingly being elucidated. Notably, FAs can induce PTMs during their metabolic processes, with one of the most recognized examples being the induction of protein acetylation via FA metabolism. Specifically, the lysine residues of histone proteins are primary targets of such acetylation [32,33]. Within chromatin, the acetylation of histones serves as a crucial factor in regulating chromatin accessibility. This can ultimately lead to epigenetic changes, significantly affecting cellular functions. In fact, ⓐ acetyl-CoA derived from lipids plays a crucial role as a primary carbon source for histone acetylation. By employing a combination of ^13^C-carbon tracing and acetyl-proteomics techniques, it has been observed that as much as 90% of the acetylation occurring on specific histone lysines originates from fatty acid carbon, even when there is an excess of glucose present [32,33]. Consequently, the excessive accumulation of cellular lipids has the potential to influence the epigenetic regulation of cells. Enzymes like ACLY and ACS, integral to FA metabolism, generate acetyl-CoA not only from FAs but also from citrate [34] and free acetate [35]. Additionally, α-ketoglutarate serves as a viable source of acetyl-CoA [36]. 

Furthermore, the oxidative stress resulting from lipid buildup can directly lead to PTMs of proteins [37]. Both oxidation and carbonylation are closely linked to oxidative stress and occur through non-enzymatic processes [38]. Among these, carbonylation, a PTM catalyzed by metals, affects lysine, proline, arginine, and threonine residues within proteins. Notably, the carbonylation of prolyl-4-hydroxylase beta (P4Hb) serves as an early target of autoimmunity and acts as a pathway that triggers an immune response involving insulin and/or proinsulin [37]. This suggests that oxidative stress induced by carbonylation may exacerbate insulin resistance in individuals with diabetes.

Additionally, while research on DKD is limited, it is essential to consider various protein lipidation processes, such as alkylation, palmitoylation, and farnesylation, as crucial links between lipotoxicity and cellular dysfunction. Palmitoylation, involving the attachment of a 16-carbon FA (palmitic acid) to cysteine residues in proteins via thioester bonds, is noteworthy [39]. Although palmitoylation is necessary for normal protein functioning, inappropriate palmitoylation resulting from lipid overload has been shown to worsen insulin resistance by disrupting the functionality of proteins like CD36 [40] and GLUT4 [41].

**Figure 1:**
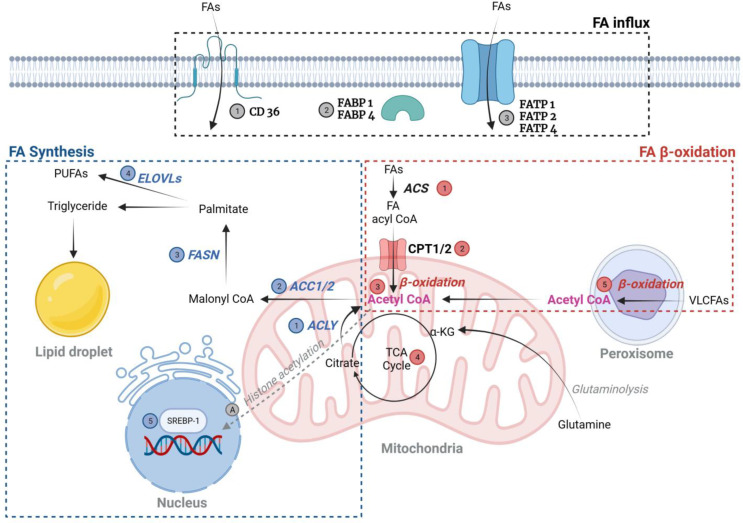
Fatty acid metabolism. FATP1, FATP2, and FATP4 primarily facilitate the transport of FFAs in tubular cells, while CD36 predominantly handles FFA transport in podocytes. Once inside the cell, these FFAs are either directed towards fatty acid oxidation (also referred to as β-oxidation) or are sequestered in the form of lipid droplets for storage. Created with BioRender.com (accessed on 8 November 2023). Abbreviations: ACC, acetyl-CoA carboxylase; ACLY, ATP citrate lyase; ACS, acyl coenzyme A synthase; α-KG, α-Ketoglutaric acid; CD36, scavenger receptor class B type I; CPT, carnitine palmitoyl transferase; ELOVLs, elongation of very-long-chain fatty acids; FABP, fatty acid-binding proteins; FASN, fatty acid synthase; FATP, fatty acid transport proteins; FFA, free fatty acid; PUFA, polyunsaturated fatty acid; VLCFA, very-long-chain fatty acid.

#### 2.1.2. Cholesterol (Figure 2)

##### Cholesterol Uptake, Synthesis, and Efflux

SREBP1 and SREBP2 have a physiological role in cholesterol synthesis. ① Initially, they move from the endoplasmic reticulum (ER) to the Golgi apparatus, undergoing a cleavage process. Subsequently, ② they relocate to the nucleus to initiate the synthesis of cholesterol. The freshly synthesized cholesterol either ③ gets converted into esterified cholesterol by sterol O-acyltransferase 1 (SOAT1) or ④ is directed to the plasma membrane for efflux through ATP-binding cassette subfamily A member 1 (ABCA1) and subfamily G member 1 (ABCG1). ⑤ The intake of cholesterol from circulating low-density lipoproteins (LDL) is overseen by the LDL receptor (LDL-R). Additionally, ⑥ the proprotein convertase subtilisin/kexin type 9 (PCSK9) aids in the degradation of LDL-R by adhering to it on the cell surface and promoting its intake into the endosomes. Finally, Niemann–Pick C1 (NPC1) located in the late lysosomes plays a crucial role in managing unesterified cholesterol levels.

Not only does lipid accumulation represent a hallmark of DKD, but so does cholesterol accumulation [42]. Regarding cholesterol synthesis, there is a notable upregulation of SREBP1 and SREBP2 in the glomeruli of DKD patients [43], with SREBP2 primarily participating in cholesterol metabolism [44]. Although attempts to alleviate albuminuria or hyperfiltration were unsuccessful, a recent investigation indicated that administering an inhibitor of SREBP1 and SREBP2 to male mice with streptozotocin (STZ)-induced diabetes mitigated the thickening of the glomerular basement membrane [45].

From the perspective of cholesterol influx, a study has shown that the genetic removal of sterol O-acyltransferase 1 in diabetic *db*/*db* mice can ease kidney damage by diminishing cholesterol esters and the buildup of lipid droplets [46]. Moreover, inhibitors of PCSK9, designed to regulate hyperlipidemia by impacting LDL absorption and elimination in liver cells, have been effective in managing the hyperlipidemia linked with nephrotic syndrome [47]. Cholesterol is primarily expelled from cells through ATP-binding cassette transporters ABCA1 and ABCG1, along with the scavenger receptor SR-BI [48]. There are findings suggesting that typical human podocytes accumulate lipid droplets and show diminished ABCA1 expression when exposed to serum from patients with both types of diabetes and early DKD [49]. Additionally, ABCA1 expression has been found to align with DKD progression indicators in both clinical settings and experimental mouse models, such as diabetic BTBR *ob*/*ob* and *db*/*db* mice [49]. Research on diabetic NOD mice unveiled a notable 48% decline in kidney ABCA1 expression [50]. The administration of ezetimibe, an ABCA1-boosting molecule, has been linked to a reduction in DKD progression and similar glomerulosclerosis symptoms [49]. Lastly, the transporters ABCG1 and SR-BI facilitate cholesterol’s movement towards mature high-density lipoprotein (HDL). In specific DKD mouse models, there was a marked decline in the expression of ABCG1 and SR-BI in mesangial and tubular cells [51].

**Figure 2:**
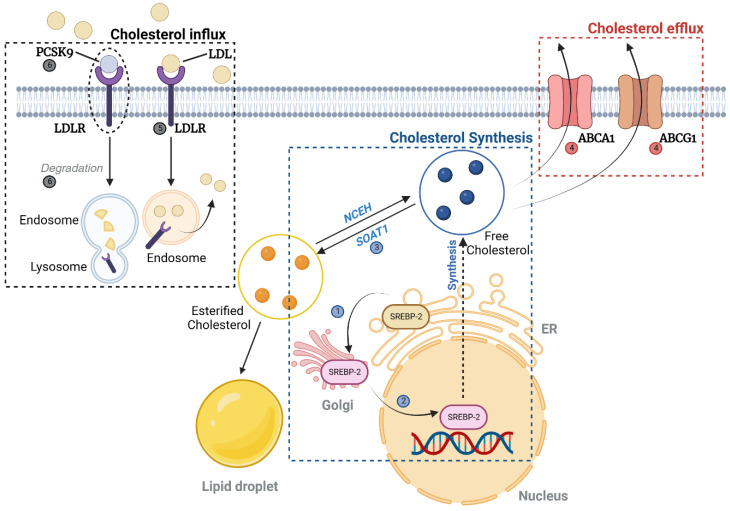
Cholesterol metabolism. SREBP2 is translocated from the endoplasmic reticulum to the Golgi apparatus, where it undergoes proteolytic cleavage. This is followed by its migration to the nucleus, where it activates the transcription of genes involved in cholesterol biosynthesis. The cholesterol thus produced is either esterified by SOAT1 or shuttled to the plasma membrane, where it can be effluxed via the action of ABCA1 and ABCG1. The uptake of cholesterol from LDL is facilitated by LDLR, while the proprotein convertase PCSK9 binds to LDLR, enhancing its endocytosis and degradation in endosomes. Created with BioRender.com (accessed on 26 November 2023). Abbreviations: ABCA1, ATP-binding cassette transporter A1; ABCG1, ATP-binding cassette transporter G1; LDL, low-density lipoprotein; LDLR, low-density lipoprotein receptor; NCEH, neutral cholesterol ester hydrolase; PCSK9, proprotein convertase subtilisin/kexin type 9; SOAT1, sterol O-acyltransferase 1; SREBP2, sterol regulatory element-binding protein 2.

### 2.2. Lipid-Induced Oxidative Stress in Lipid-Rich Kidney Disease, Including DKD

An overload of lipid can induce oxidative stress, contributing to DKD [52]. Palmitate, which is the saturated FFA, attenuated the expression of SIRT3 in murine renal proximal tubular cells. This was because the *SIRT3* KO reduced mitochondrial oxidative function and the expression of superoxide dismutase (SOD) [53]. Palmitate also induced ROS formation in human kidney 2 (HK2) cells [54], mesangial cells, and podocytes [55]. Additionally, experimental studies involving animals have demonstrated that lipid accumulation in the kidneys precipitates oxidative stress. Spontaneously hypertensive male rats and Wistar–Kyoto rats, when subjected to a high-fat diet (HFD), exhibited elevated serum FFA levels along with renal impairment, as evidenced by albuminuria and glomerular hypertrophy. Coinciding with this increased lipid accrual in renal tissues, there was a noted downregulation of SOD2 and B-cell lymphoma 2 (Bcl-2), which was orchestrated through the attenuated activity of PPARα. This biochemical cascade led to a surge in ROS and consequent oxidative stress [56]. Furthermore, this mechanism was again proven when fenofibrate treatment on *db*/*db* mice showed restored a BCL-2/BAX ratio and SOD level, and made a recovery from oxidative stress and apoptosis [57]. 

ER stress and mitochondrial dysfunction are well-recognized contributors to the onset of oxidative stress [58,59]. Interestingly, both are the important mechanisms of lipid-induced toxicity [60]. In fact, lipotoxicity has been found to induce apoptosis in mesangial cells through the activation of the protein kinase RNA-like endoplasmic reticulum kinase (PERK) and activating transcription factor 6 (ATF6) signaling pathways, which was diminished via a *PRMT1* knock down. This phenomenon is also noticed in a HFD mice model [61]. In obesity-induced mice, which are a good model for observing lipid toxicity, enhancing hepatocyte growth factor/c-Met signaling with mesenchymal stem cells led to the amelioration of tubular damage induced by palmitate and the renal damage induced by the HFD [62]. Moreover, in a mouse model of DKD, tubular cells in *db*/*db* mice displayed mitochondrial anomalies, including impaired mitophagy, excessive mitochondrial ROS production, and mitochondrial fragmentation. These cellular disturbances were observed along with a diminished expression of PINK and Parkin proteins, as well as heightened apoptotic activity [63]. Additionally, a study utilizing a diabetic mouse model revealed a notable reduction in the renal tissue levels of phosphorylated AMP-activated protein kinase (AMPK), Pink1, Parkin, LC3-II, and Atg5 in diabetic mice. The disrupted mitophagy and elevated expression of NLRP3, alongside deteriorated kidney function, proteinuria, and fibrosis, were ameliorated following the administration of Metformin, a known activator of AMPK [64].

## 3. Hypoxia and DKD

### 3.1. Increased Risk of Hypoxic Injury in Kidney

The kidney exhibits several unique characteristics in terms of oxygenation that lend credence to the hypothesis of renal arterio-venous oxygen shunting. This theory suggests that the oxygen supplied to the kidney can bypass the renal parenchyma [65], a notion substantiated via several observations. First, the close proximity and parallel orientation of the renal artery and vein allow for potential oxygen diffusion between them, leading to the possibility of oxygen shunting [66]. Secondly, despite the renal artery accessing the kidney via the renal pedicle, oxygen tension is notably higher in the cortex than that in the medulla. Given the vigorous solute reabsorption in the cortex’s proximal tubule, a region with a high ATP demand, raised oxygen tension is expected. Notably, the renal vein, which is known to have higher oxygen tension than that in the renowned oxygen-rich renal cortex [67], further bolsters the hypothesis of intra-renal oxygen shunting. Additionally, the fact that renal oxygen tension remains relatively stable even when renal blood flow fluctuates by about 30% suggests the potential existence of renal oxygen shunting [68].

Attributed to this oxygen shunting, kidneys exhibit a decrease in oxygen tension even with a slight reduction in hematocrit during normal conditions [69]. Due to this shunting, the medulla, which receives only about 10% of blood flow compared to that to the cortex, is at an increased risk of hypoxia [70]. Indeed, the oxygen tension in the medulla ranges from 10 to 20 mmHg, essentially placing it under hypoxic conditions [71]. Moreover, in the medulla, the close proximity of ascending and descending flows in the vasa recta allows for theoretical oxygen shunting [72]. Despite being inherently vulnerable to hypoxia, kidneys have their protection methods. For example, the medulla, which is more hypoxic than is the cortex, is relatively less sensitive to substances like angiotensin II and endothelin-1, which constrict blood vessels [70]. Also, erythropoietin (EPO) is secreted from the kidney. Nonetheless, these adaptations are not sufficiently robust to wholly compensate for potential hypoxic injury [72]. 

### 3.2. Renal Hypoxia and Oxidative Stress in DKD

**Figure 3:**
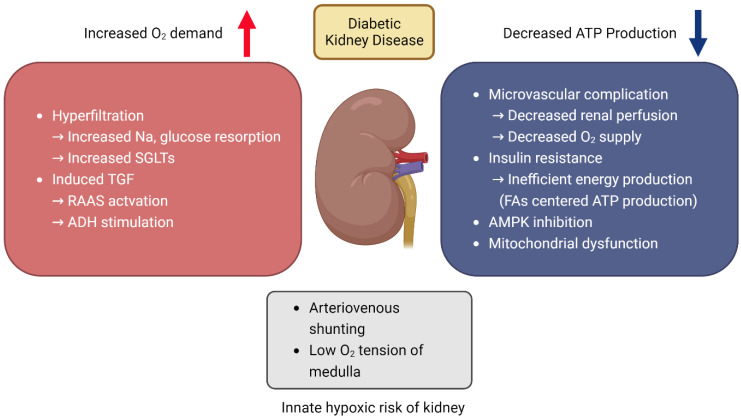
Diabetic kidney disease (DKD) aggravating renal hypoxia by increasing O_2_ demand and decreasing adenosine triphosphate (ATP) production. While inherently prone to hypoxia via arteriovenous shunting, the kidneys under DKD stress undergo hyperfiltration, increasing the ATP demand for glucose and sodium reabsorption, leading to tubular hypertrophy and upregulated SGLT. This escalates sodium reuptake, precipitating TGF and the consequent activation of the RAAS and ADH secretion. Concurrently, DKD impairs ATP synthesis due to diabetes-induced microvascular injury, which disrupts oxygen supply and renal blood flow. Insulin resistance further impedes glucose-based ATP production, necessitating a compensatory increase in FAO for energy. Additionally, DKD correlates with suppressed AMPK function and mitochondrial dysfunction, exacerbated by significant lipotoxicity. Created with BioRender.com (accessed on 7 November 2023). Abbreviations: ADH, anti-diuretic hormone; AMPK, adenosine monophosphate-activated protein kinase; FA, fatty acid; FAO, fatty acid oxidation; RAAS, renin–angiotensin–aldosterone system; SGLT, sodium–glucose cotransporter; TGF, tubuloglomerular feedback.

Renal hypoxia in DKD has been extensively documented. Since 1994, when an increase in oxygen demand in the renal tubules was first reported in rats induced with diabetes using STZ [73], a decrease in O_2_ perfusion to the renal tissue has also been verified in diabetic animal experiments [74]. Increased Na^+^/K^+^-ATPase activity in the renal cortex has been observed not only in type 1 diabetes mellitus models, such as the streptozotocin (STZ)-induced diabetic model, but also in the Zucker diabetic fatty (ZDF) rat, a model for type 2 diabetes mellitus. This increase is likely attributable to hyperglycemia and the subsequent high glucose load in the proximal tubules [75]. Along with this, such intrarenal hypoxia has been observed in mouse experiments to trigger the onset of DKD, manifesting symptoms such as proteinuria [76]. Using blood oxygenation level-dependent magnetic resonance imaging (BOLD-MRI), non-invasive studies have also shown that renal hypoxia manifests early in diabetes and worsens over time [77,78]. Additionally, BOLD-MRI showed that patients with DKD had lower oxygen tension in the renal cortex compared to healthy adults, but the medulla’s hypoxia was less severe, possibly due to a significant decline in tubule function and reduced ATP demand [79]. The kidney, which is highly vulnerable to hypoxia, experiences a growing disparity between renal ATP demand and generation during DKD [72] (Figure 3), exacerbating hypoxic injury, ultimately promoting the progression of the disease [80]. 

When DKD occurs, the kidney undergoes hyperfiltration and is obliged to reabsorb the filtered glucose and sodium. This leads to tubular growth and an increase in the number of SGLTs, which in turn demands higher ATP consumption for the reabsorption of glucose and sodium. Additionally, this accelerated sodium reabsorption is perceived by the thick ascending limb’s macula densa as a decrease in tubular sodium, thereby inducing tubuloglomerular feedback (TGF). This feedback mechanism, by modulating the tone of the afferent and efferent arterioles [81], intensifies glomerular filtration, establishing a deleterious feedback loop. Furthermore, this mechanism stimulates the renin-angiotensin-aldosterone system (RAAS) [82]. According to one study, when these situations are simulated using computer models, the oxygen demand of the kidneys increases by 50% to 100% [83].

Conversely, the body’s ATP generation capacity is significantly diminished due to DKD. Primarily, since DKD results from diabetes-induced microvascular damage [84], there are issues with oxygen delivery due to compromised renal perfusion. Moreover, due to insulin resistance, the efficient ATP production pathway utilizing glucose is undermined, causing a shift towards reliance on FAO for ATP synthesis [85,86]. This is considerably less efficient in energy production. Furthermore, renal hypoxia inhibits AMPK, impending ATP synthesis [87], and pronounced lipotoxicity in DKD, leading to mitochondrial dysfunction, is well documented [88]. Considering the importance of mitochondrial oxidative phosphorylation in ATP synthesis, this can be detrimental to ATP production.

Oxidative stress, which patients with DKD are especially prone to [89], refers to the condition where the production of ROS exceeds the capacity of the anti-oxidant system to neutralize them, leading to a deterioration in cellular and tissue functions [90]. Hypoxia alters the function of the cytochrome chain involved in mitochondrial oxidative phosphorylation. This leads to reduced ATP production and heightened ROS levels [91], coupled with the diminished activity of the cell’s anti-oxidant defenses [92], potentially culminating in oxidative stress. Conversely, there are claims that oxidative stress induced in kidney disease situations can lead to hypoxia. Notably, the well-known uremic toxin, indoxyl sulfate, has been reported to provoke oxidative stress, resulting in increased oxygen consumption and subsequent hypoxia [93]. The relationship between chronic hypoxia and oxidative stress can thus be dynamic, with each potentially serving as both a cause and an effect at different times. The emergence of a vicious cycle due to this interplay is entirely conceivable.

### 3.3. HIF-1 Activation in Hypoxia: A Double-Edged Sword

The HIF-1 protein consists of α and β subunits [94]. While the β subunit is relatively stable and constantly present, the α subunit is not [95]. Among the α variants, namely 1, 2, and 3, most research has been conducted on types 1 and 2 [96]. In the kidney, HIF-1α is predominantly expressed in tubular epithelial cells, while HIF-2α is primarily found in endothelial cells and interstitial cells [97]. When the α and β subunits of HIF bind, they enter the nucleus and induce the expression of target genes. It is known that the downstream targets of HIF-1 include genes involved in iron regulation, glycolysis, cell survival, erythropoiesis, and angiogenesis [98]. Under typical conditions, the regulation of HIF-1 involves its α subunit being targeted for ubiquitination by the von Hippel–Lindau protein E3 ubiquitin ligase, due to the action of prolyl hydroxylase (PHD) [99]. This usually results in its degradation. However, under hypoxic conditions, PHD loses its function, preventing the ubiquitination of the HIF-1α subunit. The stabilized HIF-1α subunit then binds with the β subunit, becoming active under hypoxia. Given the kidney’s unique perfusion structure, oxygen shunting, and exacerbated renal hypoxia in DKD, the HIF-1 downstream pathway aids cells in managing hypoxia by decreasing cellular oxygen consumption while increasing glycolysis. One mechanism of the HIF-1 pathway in response to hypoxia involves the upregulation of pyruvate dehydrogenase kinase 1 by HIF, which inhibits pyruvate dehydrogenase. This diverts pyruvate from the TCA cycle and oxidative phosphorylation, favoring the glycolytic pathway [100]. The suppression of oxidative phosphorylation under hypoxia can also reduce ROS production, thereby mitigating oxidative stress [101]. Furthermore, HIF-1 inhibits the activity of complex 1 in the electron transport system [102] and alters the composition of a cytochrome c oxidase [103]; these manipulations can decrease oxidative stress.

While HIF-1 activation has benefits such as promoting glycolysis, it also suppresses carnitine palmitoyl transferase A1 [104], reducing the transport of FAs into the mitochondria and diminishing FAO [100]. In the short term, this reduces oxygen consumption and can mitigate ROS production and oxidative stress induced by hypoxia. However, in the long run, it may exacerbate lipotoxicity. Additionally, HIF-1α is known to be involved in M1 polarization [105], a proinflammatory macrophage phenotype, and is associated with Th17 differentiation in CD4^+^ T cells [106] and effector differentiation in CD8^+^ T cells [107], potentially leading to inflammation. Therefore, the role of HIF proteins in kidney diseases should be considered in context.

## 4. Oxidative Stress and DKD

### 4.1. The Role of ROS and Oxidative Stress in DKD

ROS have a pivotal role in various cellular functions, including cell development, differentiation, damage, senescence, and apoptosis. They can also induce DNA harm, impact hormonal responses, control ion channel operations, and influence glucose processing [108].In terms of the ‘kidney’, ROS can exacerbate renal disorders through multiple pathways. It may inflict direct harm to renal cells, which results in reduced renal function, promotes inflammation, and causes fibrosis. Furthermore, it has the capacity to trigger signaling pathways and activate transcription factors that intensify these conditions. Conditions such as diabetes, high blood pressure, and metabolic syndrome are linked with increased oxidative stress within the kidneys and a disruption in the normal functioning of NOS, leading to a disproportionate ratio of ROS to nitric oxide (NO) [109,110,111]. A surge in ROS might stem from increased creation, a breakdown in anti-oxidant defenses, or a combination of the two [112]. Consequently, on a broader scope, oxidative stress instigates inflammation, renal fibrosis, and other pathological transformations, culminating in decreased GFR and advancing DKD [113]. However, we should remember that while an excess of ROS can cause harm, a minimal amount is crucial for physiological functions, like in standard renal functions [113]. Notably, ROS production diminishes in mice treated with STZ, a well-known diabetes model [112]. This highlights the dual role of ROS as both a harmful and beneficial agent. 

### 4.2. ROS, Oxidative Stress, and Intracellular Signaling Pathways

Beyond the previously mentioned HIF-1 pathway, there are intracellular signaling pathways induced by oxidative stress. Understanding these pathways is critical in identifying therapeutic targets for diseases predominantly affected by oxidative stress.

#### 4.2.1. Keap1-Nrf2 Pathway

Kelch-like erythroid cell-derived protein with the CNC homology-associated protein 1 (Keap1)-Nrf2 pathway is an important form of cellular signaling for reducing oxidative stress. Nrf2 is a basic leucin zipper-type transcription factor. Nrf2 binds with Keap1, and this complex undergoes degradation via ubiquitination. Therefore, Nrf2 exists in a relatively low concentration in its steady state. However, when oxidative stress occurs, it induces modifications in the cystine residues of Keap1, which results in the dissociation of Nrf2 from Keap1. The liberated Nrf2 then translocates to the nucleus, where it associates with small musculo-aponeurotic fibrosarcoma oncogene homologues (sMaf) and anti-oxidant responsive elements (ARE), initiating the transcription of components of the redox system that counteract oxidative stress, such as glutathione synthase and heme oxygenase 1 (HO1) [114]. For this reason, the Keap1-Nrf2 signaling pathway has been widely recognized as a therapeutic target for combating oxidative stress. Indeed, the efficacy of Nrf2 in mitigating oxidative stress has been reported not only in ischemic reperfusion injury [115] and unilateral ureter obstruction models but also in diabetic mouse models [116], where it has been observed to slow the progression of renal disease [117].

#### 4.2.2. Forkhead Box O (FoxO) Proteins

FoxO is primarily known as a protein inhibited by the phosphatidylinositol 3-kinase (PI3K)/Akt signaling pathway, which plays a crucial role in regulating insulin sensitivity in target tissues, including the kidney. This is part of a family of transcription factors in mammals comprising FoxO1, FoxO3A, FoxO4, and FoxO6. These proteins are significant in the regulation of aging, cellular metabolism, insulin sensitivity, and lifespan [118]. Recently, it has been found to also function as a redox molecule. When oxidative stress arises, FoxO proteins bind to transportin transporters and subsequently relocate into the nucleus. Once inside, they function as transcription factors, initiating the transcription of genes associated with the anti-oxidant response [119]. FoxO1 is abundantly found in tissues that are sensitive to insulin, which includes the liver, fat, muscle, and pancreatic tissue. It is considered a critical overseer of insulin signaling and the equilibrium of glucose levels, due to its role in orchestrating gene expression networks that oversee glucose utilization. Recent studies showed that FoxO1 and FoxO3 were decreased in the kidney of diabetic mice and rats, which was related to an increase in oxidative stress [120,121], respectively, which was reversed via resveratrol treatment. Research indicates that certain genetic alterations in FOXO1 could potentially heighten the susceptibility to DKD [122].

#### 4.2.3. Nuclear Factor (NF)-κB Pathway

Distinct from the two pathways previously discussed, NF-κB is a well-established member of the proinflammatory pathway. Typically, it remains in an inactive state due to its association with I-κB. However, under conditions of oxidative stress, IκK mediates the phosphorylation of I-κB, liberating NF-κB to translocate into the nucleus, where it initiates the transcription of genes related to inflammation and cellular growth and survival [95]. It is known that this NF-κB pathway can become more activated by increased ROS, particularly when the Nrf2 pathway is compromised [123]. 

### 4.3. ROS, Oxidative Stress, and Cellular Organelles in the Context of DKD

In the kidney, ROS are produced through the participation of enzyme groups like NADPH oxidases (NOX) and within specific cellular components like the ER, peroxisomes, and mitochondria [124]. Notably, mitochondria serve as the principal site for ROS generation, stemming from the redox reactions occurring in its electron transport chain [112]. In fact, mitochondrial density in the kidney is the second highest in the human body, following that in the heart [124]. Given that the kidneys are rich in mitochondria, a primary source of ROS, it is essential to elucidate the relationship between renal cellular organelles and oxidative stress.

#### 4.3.1. Mitochondria

Due to premature release of protons at complex I and III of the electron transport chain, mitochondria generate ROS. When these ROS are not adequately neutralized, oxidative stress ensues. Since DKD is widely recognized as a condition influenced by ROS dysregulation, it is reasonable to propose that mitochondrial function plays a crucial role in the progression of DKD. Indeed, there have been reports indicating that DKD compromises various essential aspects of mitochondrial functionality, encompassing mitochondrial biogenesis, as well as the dynamics of mitochondrial fission and fusion [125]. Investigations into the role of mitochondria in DKD have yielded intriguing findings. For instance, mice deficient in dynamin-related protein-1 (DRP1), a protein implicated in mitochondrial fission and fragmentation [126,127], exhibit a suppression of DKD progression upon the induction of diabetes [128]. Correspondingly, studies employing inhibitors of DRP1 have demonstrated similar therapeutic effects in the context of DKD [129].

Mitophagy, the process by which damaged mitochondria are cleared from the cell, is vital for maintaining normal mitochondrial function within cells. Key players in mitophagy, PTEN-induced kinase 1 (PINK1), and Parkin, which facilitate the ubiquitination and degradation of damaged mitochondria, are found to be diminished in DKD [63]. Additionally, the expression of optineurin, which is essential for the formation of mitophagosomes, is also reduced in the tubular cells of diabetic patients [130].

Mitochondrial biogenesis is critical for the proper functioning of healthy cells, with PGC-1α being a significant contributor to this process and to recovery from kidney injury [131]. Numerous reports have corroborated the presence of mitochondrial dysfunction in DKD [124]. Therefore, it appears that future research should focus on restoring normal mitochondrial function as a therapeutic target to address the oxidative stress caused by DKD.

#### 4.3.2. ER and Peroxisome

The ER is responsible for the proper folding and post-translational modification of membrane and secretory proteins. When these processes are disrupted, resulting in the accumulation of improperly formed proteins within the ER, it triggers a condition known as ER stress. This ER stress plays a role in the pathology of DKD within certain cultured cellular models, and the unfolded protein response (UPR) pathway becomes engaged in response to high levels of glucose, lipids, and advanced glycation end-products (AGEs). In mice models with diabetes induced by STZ, there is an upsurge in ER stress markers along with an amplification of apoptosis in the cells of the glomeruli and tubules [132]. ER stress appears to have a reciprocal influence with oxidative stress [133]. Within the ER, disulfide bonding, which is pivotal for protein folding, occurs frequently. However, the formation of protein disulfide bonds is known to generate ROS within the ER, and it is believed that when this production exceeds the capacity of the ER’s redox system, oxidative stress ensues [134,135]. Conversely, ROS can also interfere with the protein folding process in the ER. Indeed, several oxidants, including peroxides, have the potential to induce aberrant UPR [136]. 

Peroxisomes are cellular organelles integral to the detoxification of ROS, equipped with enzymes such as SOD, glutathione peroxidase, and catalase, with the peroxisomes accounting for approximately 90% of the cell’s catalase [137]. The peroxisomal membrane protein PEX14 plays a crucial role during cell division by increasing catalase levels, thus safeguarding the exposed DNA from ROS damage during cellular division [138]. Scientific research reports indicate that peroxisomal dysfunctions can contribute to the development of DKD [30].

#### 4.3.3. Mitochondria-Associated ER Membrane (MAM)

Previous research has suggested that cell organelles like the mitochondria and ER operate independently. However, recent findings support the notion that these organelles do not function in isolation. Research has revealed the existence of a physical connection between the ER and mitochondria known as the mitochondria-associated membranes (MAM) [139]. The MAM plays a multifaceted role in cellular operations, encompassing lipid metabolism, the modulation of calcium signals, responses to ER stress, mitochondrial performance, and the processes of cell death and self-digestion [140]. In terms of DKD, there is a noted decrease in the levels of the anti-oxidant enzyme DsbA-L, which is crucial for redox reactions, alongside a diminished capacity for sustaining the integrity of the MAM structures in DKD [141]. 

## 5. Therapeutic Approaches to Modify DKD

### 5.1. Antilipidemic Drug 

#### 5.1.1. Statins/Fenofibrate

Statins, which function as 3-hydroxy-3-methylglurtaryl coenzyme A reductase inhibitors, are among the most prescribed medications globally for the management of dyslipidemia. While statins have been shown to decrease cardiovascular complications in patients with CKD, their advantageous impact on eGFR remains ambiguous [142]. Indeed, a meta-analysis focusing on CKD patients revealed that within the DKD subset, statins did not exert any effect on eGFR [143]. Fenofibrate is PPARα agonist that is used for treating dyslipidemia. Fenofibrate showed a significant effect on dyslipidemia even in general populations [144], but there is no significant amount of evidence that fenofibrate can deter CKD progression. While it has been reported that pemafibrate increases eGFR, this finding is based on a study of only 16 patients [145]. Furthermore, in DM patients, fenofibrate improved the state of albuminuria, but did not show an effect on serum creatinine levels [146]. However, with respect to FA metabolism and oxidative stress, pemafibrate has been reported to protect the kidneys from FA-induced nephropathy by preserving renal FA metabolic processes [147].

#### 5.1.2. Ezetimibe

Ezetimibe, designed to inhibit the absorption of cholesterol by targeting NPC1L, can, when paired with a statin, decrease the incidence of cardiovascular events in individuals with CKD [148]. Ezetimibe was particularly effective in reducing renal parenchymal fat content, especially in patients with elevated renal fat concentrations, and particularly those with DKD [149].

#### 5.1.3. PCSK9 Inhibitors

PCSK9, which is a monoclonal antibody for targeting PCSK9, disturbs the binding of LDL-R with LDL. Thus, PCSK9 inhibitors are considered a supplementary treatment option for dyslipidemia. As expected, alirocumab and evolocumab reduced serum LDL successfully in CKD patients [150,151]. In experimental settings, PCSK9 inhibitors have been shown to diminish renal lipotoxicity induced by diet, specifically by decreasing the expression of CD36 on the cell surface [152]. This effect is related to the management of lipid influx.

#### 5.1.4. ABCA1 Inducer

ABCA1 is a transporter that facilitates the transport of cholesterol outside of the cells. The dysfunction of ABCA1 can aggravate renal injury in DKD, contributing to inflammatory injury and the apoptosis of glomerular endothelial cells. This is due to ER stress resulting from the accumulation of cholesterol [153], which also manifests as podocyte injury and decreased cardiolipin oxidation [154]. When ABCA1 inducer A30 was treated on *db*/*db* mice, the effacement of the foot process of podocytes, albuminuria, and oxidative stress were recovered [49].

### 5.2. SGLT2 Inhibitor/GLP-1 Agonist

#### 5.2.1. SGLT2 Inhibitor

SGLT2 inhibitors have risen to prominence as a cornerstone in the therapeutic landscape for DKD [155], with emerging reports substantiating their efficacy in treating not only DKD but also non-diabetic renal disorders. Moreover, a growing body of research suggests their contributory role in attenuating lipotoxicity. Studies have demonstrated that dapagliflozin mitigates lipid accumulation in the renal tubules of HFD mice [156] and engenders similar lipid stress-inhibitory effects in *db*/*db* mice, thereby suppressing ER stress [157]. Canagliflozin and Ipragliflozin are also reported to preserve mitochondrial function and facilitate FA metabolism in diabetic mouse models [158,159].

Remarkably, recent discourse posits that SGLT2 inhibitors may ameliorate renal hypoxia exacerbated by DKD. The administration of these inhibitors is associated with an initial dip in eGFR, a phenomenon attributed to the TGF mechanism, triggered by the increased detection of sodium and glucose in the tubular lumen by the macula densa [160,161]. This reduction in GFR is anticipated to diminish the tubular workload and decrease oxygen demand. A prediction supported by a study utilizing mathematical modeling projected up to a 30% reduction in renal oxygen demand resulting from SGLT2 inhibition [162]. Furthermore, in type 1 diabetic mouse models, SGLT inhibition has been shown to curtail hyperfiltration and the activity of the Na^+^/K^+^-ATPase pump [162], as well as sodium reabsorption mechanisms well known to exacerbate renal hypoxia in DKD. Additionally, SGLT2 inhibition has been noted to improve cortical hypoxia in a type 1 DM rat model [163], while also normalizing TCA cycle intermediates in type 2 DM, leading to a reduction in oxidative stress and proteinuria [164]. In corroborating studies, a reduction in the expression of HIF-1α in proximal tubular epithelial cells was observed upon treatment with SGLT2 inhibitors, suggesting an amelioration of tubular cell hypoxia consequent to the therapy [165].

#### 5.2.2. GLP-1 Agonist

The GLP-1 agonist also seems to have some benefits in terms of DKD [166,167]. However, its association with lipotoxicity requires further investigation. In one animal study, liraglutide administered over 12 weeks enhanced the lipid profiles in the plasma and diminished the accumulation of lipid droplets in the proximal tubules within a rat model of DKD [168]. Also, in terms of cholesterol efflux, the GLP-1 agonist was found to upregulate ABCA1 expression in many organs including kidney glomerular endothelial cells [169].

### 5.3. NRF2 Activators

#### 5.3.1. Bardoxolone Methyl

Bardoxolone methyl, a derivative of a triterpenoid compound, enhances the anti-oxidant response while concurrently inhibiting pro-inflammatory pathways. This action serves to diminish oxidative stress and inflammation, thereby supporting mitochondrial function [170]. Multiple clinical studies involving patients with type 2 diabetes and CKD have examined bardoxolone methyl. These trials have documented enhancements in GFR, as determined by inulin clearance, creatinine clearance, and eGFR, following treatment with bardoxolone methyl [171,172,173]. 

Despite its remarkable efficacy in enhancing renal function, bardoxolone methyl has exhibited significant safety concerns. Reports have emerged of albuminuria, anorexia, hypomagnesemia, volume overload, and elevated blood pressure [171]. The phase 3 BEACON trial [173], which included participants with grade 4 DKD, was prematurely terminated due to a significant increase in the risk of hospitalizations or death from heart failure. A subsequent analysis of the trial indicated that the critical heart failure events primarily occurred within the first four weeks of treatment [174]. Post hoc analysis led to the identification of elevated baseline B-type natriuretic peptide levels and prior hospitalization for heart failure as risk factors for drug-induced cardiac events [175]. As a result, subsequent clinical investigations, which excluded patients with these risk factors, began, focusing particularly on individuals with DKD in Japan. Through the phase 2 TSUBAKI trial [176] and the phase 3 AYAME trial [177], bardoxolone methyl demonstrated improvements in GFR among DKD patients. Therefore, the potential of bardoxolone methyl as a therapeutic agent for DKD remains a subject of research, necessitating further studies to elucidate its viability and safety profile.

#### 5.3.2. Curcumin

Curcumin, a culinary spice belonging to the polyphenol family, instigates the NRF2/KEAP1/ARE pathway by facilitating the dissociation of NRF2 from KEAP1 [178]. This activation results in the production of anti-oxidant enzymes. Consequently, curcumin is recognized for its capacity to mitigate oxidative stress, reduce inflammation, modulate lipid metabolism, and regulate the immune response [179,180]. Nonetheless, its inherent instability and limited systemic availability present significant challenges to its therapeutic application [181]. To overcome these obstacles, a derivative known as C66-curcumin ((2E,6E)-2,6-bis[2-(trifluoromethyl)benzylidene]cyclohexanone) has been synthesized [182]. Intriguingly, C66-curcumin has hinted at potential alternative mechanisms for alleviating oxidative stress, going beyond the NRF2/KEAP1/ARE pathway [183].

In 2021, a meta-analysis encompassing five randomized controlled trials (RCTs) with 290 participants suffering from DKD in total was conducted. This analysis conclusively demonstrated that curcumin supplementation significantly enhances serum creatinine, total cholesterol, diastolic blood pressure, and fasting blood glucose levels in DKD patients, as supported by a moderate degree of certainty in the evidence [179]. Consequently, comprehensive research is warranted to translate the promising therapeutic potential of curcumin into an effective treatment approach for DKD.

#### 5.3.3. Sulforaphane

Isothiocyanates constitute a class of natural compounds rich in glucosinolates, which attain their bioactive state upon hydrolysis by the myrosinase enzyme [184]. Sulforaphane stands out as the most renowned isothiocyanate. While sulforaphane has demonstrated acceptable safety profiles and relatively favorable bioavailability, its stability is excessive for therapeutic applications [185]. Despite the synthesis of several analogues, none have surpassed the efficacy of the original compound. In the context of DKD, sulforaphane activates the NRF2/KEAP1/ARE pathway through the stimulation of NRF2 [186]. Numerous animal studies have indicated the potential of sulforaphane as a preventive measure against the progression of DKD [186,187]. However, the limited availability of human clinical studies presents a challenge in definitively establishing the impact of sulforaphane on DKD [188].

### 5.4. Resveratrol

Resveratrol, a polyphenol compound primarily found in grapes and red wines, acts as an immediate anti-oxidant. It neutralizes ROS and reactive nitrogen species (RNS) along with their subsequent organic radicals, modulates intracellular anti-oxidant mechanisms, and helps maintain a balance in the cellular oxidation-reduction status [189]. It is reported to mitigate several pathologic mechanisms associated with diabetes, such as FA synthesis, inflammation, and oxidative stress [190,191]. Although it was not used in the exact same diabetes mouse model, resveratrol reduced blood glucose levels, improved serum lipid profiles, and improved renal function in HFD mice [192]. Furthermore, there is an association between resveratrol and adiponectin. Resveratrol increased adiponectin levels in the serum, decreased proteinuria, and reduced glomerular mesangial thickening and apoptotic cell death within the glomeruli of *db/db* mice [193]. Although resveratrol reduced albuminuria in a human study, there was no improvement in serum creatinine levels. Further studies are still required to fully understand the effect of resveratrol on DKD [189].

### 5.5. Vitamin D

According to some reports, vitamin D has the potential to reduce oxidative stress in diabetes. The supplementation of vitamin D3 has been shown to enhance anti-oxidant activity and decrease oxidative stress [194]. Additionally, the vitamin D receptor agonist has been found to decrease Pin1 protein, resulting in the inhibition of mitochondrial oxidative stress and inflammation [195]. In terms of DKD, the approach to managing DKD that combines vitamin D supplementation with standard DM control and RAS inhibition has shown a notable decrease in albuminuria [196]. However, the effectiveness of vitamin D supplementation in DKD patients with low vitamin D levels remains a topic of debate [197]. A comprehensive systematic review and meta-analysis, examining the impact of various forms of vitamin D on DKD patients, found that calcitriol, alfacalcidol, and vitamin D3 effectively lowered urinary protein excretion and key inflammatory markers including TNF-α, IL-6, and C-reactive protein. Nevertheless, these treatments did not significantly affect serum creatinine, eGFR, or blood glucose control [198].

### 5.6. Adiponectin Receptor Activator

Adiponectin is an adipokine secreted by adipocytes, and it is well recognized for its role in regulating glucose levels, lipid processing, and insulin responsiveness due to its anti-inflammatory and anti-oxidative properties [199]. It is involved in FA metabolism through the AMPK pathway and PPARα expression. The adiponectin receptor is widely expressed but decreased in the glomerulus of DKD patients [200]. AdipoRon, an adiponectin receptor agonist [201], significantly reduced albuminuria in *db*/*db* mice by enhancing AMPK activity and PPARα expression, along with their downstream signaling related to anti-oxidative, anti-inflammatory, and lipid metabolism. Improvements in diabetes-induced oxidative stress and the inhibition of apoptosis in the kidneys, induced by AdipoRon, have been observed to ameliorate relevant intracellular pathways linked to lipid accumulation. The administration of AdipoRon in *db*/*db* mice reinstated the phosphorylation of AMPK and the expression of PPARα, and concurrently downregulated acetyl-CoA carboxylase, a pivotal enzyme in FA synthesis, as well as SREBP-1c, a transcription factor instrumental in lipid synthesis [200].

### 5.7. HIF-1 Stabilizer (Prolyl Hydroxylase Inhibitor)

Prolyl hydroxylase inhibitors function by inhibiting the activity of proteins that degrade HIF. This inhibition stabilizes HIF, bypassing its normal degradation under regular oxygen tension, thereby activating endogenous erythropoiesis and enhancing the production of red blood cells [202]. HIF stabilizers, including compounds like roxadustat, vadadustat [203,204], daprodustat [205,206], and molidustat have been proposed for clinical research, primarily targeting the treatment of renal anemia [207]. Their oral administration offers a significant advantage, and they have demonstrated efficacy in improving anemic indices, particularly in patients with CKD not dependent on dialysis [207]. Consequently, it is reasonable to anticipate that these agents could have applications extending beyond the treatment of renal anemia serving as adjunct therapies in DKD by ameliorating renal hypoxia.

As expected, beyond their use in anemia treatment, there are reports indicating the potential of HIF stabilizers, like enarodustat, in the treatment of early-stage DKD [208]. In one study, enarodustat exhibited therapeutic potential in an STZ-induced diabetes mouse model by attenuating FA and amino acid metabolism and enhancing glucose metabolism, thereby restoring the metabolic alterations induced by insulin resistance. Moreover, it also demonstrated efficacy in alleviating oxidative stress in renal tissues [208]. Therefore, further research is warranted to explore the significant potential of HIF stabilizers in halting the progression of DKD.

### 5.8. Potential Concerns in Applying Anti-Oxidants for Treating DKD

Anti-oxidants, as potential therapeutic agents for DKD, harbor considerable promise, as previously discussed. In 2017, a meta-analysis encompassing 14 randomized controlled trials (RCTs) examined the efficacy of anti-oxidant treatment in DKD, concluding that anti-oxidants might be beneficial in the early phases of DKD [209]. However, this study noted the small sample sizes of most research and the difficulty in assessing hard endpoints such as end-stage renal failure [209]. A more recent meta-analysis, including 19 RCTs, observed improvements in glycated hemoglobin and the urine albumin to creatinine ratio in DKD patients with anti-oxidant use [210]. Yet, the analysis also highlighted the limitation of insufficient sample sizes in conducted studies. Additionally, of the 19 studies included in this meta-analysis, adverse events were reported in 6. Therefore, despite oxidative stress playing a crucial role in the pathogenesis of DKD and the apparent importance of anti-oxidants as potential therapeutic agents, the possibility of adverse effects must be considered [210]. For instance, in a study involving male Wistar rats administered 10% fructose-enriched drinking water, notable increases in body weight, plasma glucose, and sodium levels, and a decline in renal properties were observed [211]. Contrary to expectations, the administration of the anti-oxidant quercetin unexpectedly exacerbated some situations including the elevation of serum creatinine levels. Furthermore, quercetin failed to normalize the activity of renal Na^+^/K^+^ ATPase, which is upregulated by the administration of a 10% fructose solution [211].

Hence, the administration of anti-oxidants requires careful consideration, and continued research is necessary for their future applications.

## 6. Conclusions and Perspectives

**Figure 4:**
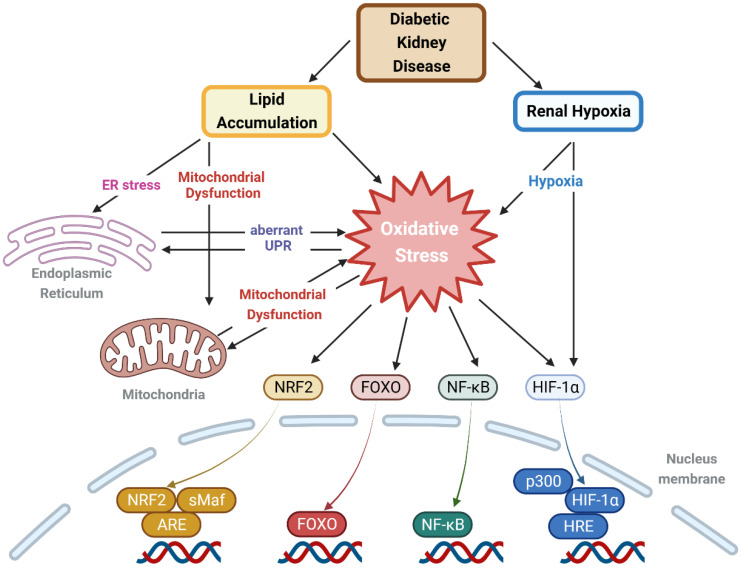
Intracellular lipid accumulation and renal hypoxia, caused by DKD, leading to cellular organelle dysfunction and ultimately inducing oxidative stress, which in turn activates various cellular signaling pathways. Lipid accumulation and renal hypoxia caused by DKD can induce oxidative stress, either directly, through ER stress, or by triggering mitochondrial dysfunction. Oxidative stress, in turn, can lead to ER and mitochondrial dysfunction, creating a reciprocal influence between organelle malfunction and oxidative stress. The resulting oxidative stress activates defensive mechanisms or inflammatory responses through pathways such as NRF2/KEAP1/ARE, the FOXO family, the NF-κB pathway, and the HIF pathway, all of which also interact with one another. Created with BioRender.com (accessed on 7 November 2023). Abbreviations: ARE, anti-oxidant response element; ER, endoplasmic reticulum; FOXO, forkhead box O; HIF, hypoxia-inducible factor; HRE, hypoxia response element; KEAP1, Kelch-like ECH-associated protein 1; NF-κB, nuclear factor kappa light chain enhancer of activated B cells; NRF2, nuclear factor erythroid 2-related factor 2; sMaf, small Maf protein; UPR, unfolded protein response.

Oxidative stress constitutes a pivotal hallmark of DKD pathology, recognized for its significant contribution to both the initiation and progression of the disease. Among the numerous factors underscoring the importance of oxidative stress in DKD, lipotoxicity and renal hypoxia are standout elements that are unique to this condition. Insulin resistance, a critical mechanism contributing to diabetes and its complications, leads to a lipogenic state, precipitating severe lipid accumulation within organs and consequent toxicity concerns. Moreover, the kidney’s distinctive anatomy and high metabolic demands make it susceptible to hypoxia, a risk exacerbated by increased ATP requirements and diminished production in diabetes, elevating the peril of renal hypoxia. Recent experimental and clinical evidence demonstrates the interaction between lipotoxicity and hypoxia, exacerbating DKD. Therefore, when considering oxidative stress as a therapeutic target for DKD, it becomes imperative to comprehend how fundamental mechanisms like lipotoxicity and renal hypoxia contribute to oxidative stress (Figure 4). This review explores these mechanisms, current pharmacological interventions, and areas warranting further research. Given that DKD represents a significant portion of renal ailments and the vital role of oxidative stress as a therapeutic target, it is incumbent upon the medical research fields to intensify efforts in studying therapies that can mitigate lipotoxicity and renal hypoxia.

**Table 1:** Overview of animal models of diabetes utilized in the studies cited in this review. Abbreviation: ApoE, apolipoprotein E; HFD, high-fat diet; NOD, non-obese diabetic; STZ, streptozotocin; ZDF, Zucker diabetic fat.

Type	Model	Advantages	Limitations	Reference Numbers in This Review
Type 1 DM	STZ	✓ Cheap and simple ✓ Absolute insulin deficiency	✓ Mild renal lesions ✓ Non-specific cytotoxicity at high dose	[49]
	Unilateral nephrectomy + STZ	✓ Faster than STZ in developing renal failure	✓ Limited role in CD1 strain ✓ Need for surgical technique	[45]
	Alloxan	✓ Popular chemical agent to establish Type 1 DM	✓ Less potency and more tissue toxicity than STZ	[76]
Type 1 DM (Auto-immune)	NOD	✓ Spontaneous autoimmunity with autoantibodies and autoreactive T cells	✓ Much more severe insulitis than human	[50]
Type 2 DM	*db*/*db*	✓ Most widely used model ✓ Severe albuminuria ✓ Severe mesangial proliferation	✓ Leptin resistance—uncommon in human DKD ✓ Immune complex deposit ✓ No progression of albuminuria	[19,23,46,49,57,63,120,128,129,157,193,200]
	*ob*/*ob*	✓ Severe mesangial proliferation ✓ The recruitment of monocytes/macrophages, consistent with human DKD	✓ Infertility ✓ Leptin deficiency—not a feature of human DKD	[49,164]
	HFD	✓ Excellent simulation for human type 2 DM	✓ Hard to develop nephropathy with a HFD alone	[55,61,156,158,159,186]
	HFD + STZ	✓ Easier to develop nephropathy than with a HFD alone	✓ Obesity and insulin resistance are less than they are with a HFD alone	[64]
	HFD + ApoE−/−	✓ Good for early-stage DKD model ✓ Good simulation for human non-albuminuric DKD	✓ Mild renal impairment ✓ Not progressive renal lesions	[169]
	ZDF	✓ Possible to develop nephropathy with metabolic syndrome and diabetes features	✓ Inducing severe hydronephrosis irrelevant to DKD ✓ Complications like renal abscess and pyelonephritis	[75]

## Data Availability

No new data were created or analyzed in this study. Data sharing is not applicable to this article.
